# Upregulation of *GZMK*, *TREM2*, and *OR4D10* as Prognostic Biomarkers in Thyroid Cancer: A Pan-Cancer and TCGA Data Analysis

**DOI:** 10.3390/ijms26083887

**Published:** 2025-04-20

**Authors:** Nuoyan Zhu, Liangliang Cai, Li Qian

**Affiliations:** Medical College, Yangzhou University, Yangzhou 225012, China; 222205162@stu.yzu.edu.cn

**Keywords:** TCGA, prognostic biomarker, thyroid cancer (THCA), immunosuppressive tumor microenvironment (iTME)

## Abstract

The study of gene anomalies linked to thyroid cancer is gaining more and more attention, and these molecular indicators can offer scholarly support for thyroid cancer diagnosis, therapy selection, and prognosis. Genotype–tissue expression pan-cancer data and The Cancer Genome Atlas (TCGA) were used to investigate the expression of *GZMK*, *TREM2*, and *OR4D10*. In order to assess the relationship between *GZMK*, *TREM2*, and *OR4D10* expression and patient outcome, TCGA clinical survival data were used. We used the clusterProfiler R software tool to conduct enrichment analysis of *GZMK*, *TREM2*, and *OR4D10*. Moreover, TCGA database analysis was used to assess the relationship between immune cell infiltration and *GZMK*, *TREM2*, and *OR4D10* expression. *GZMK*, *TREM2*, and *OR4D10* were strongly expressed in several kinds of malignancies including thyroid cancer. Gene sets related to proliferation that are involved in leukocyte cell–cell adhesion and mononuclear cell differentiation were significantly correlated with high expression of *GZMK*, *TREM2*, and *OR4D10*. Additional investigation revealed a correlation between high T cell and DC (dendritic cell) infiltration scores and high expression of *GZMK*, *TREM2*, and *OR4D10*. According to our research, *OR4D10*, *TREM2*, and *GZMK* could all be genes associated with thyroid cancer.

## 1. Introduction

Cancer, a complex and heterogeneous group of diseases, remains a significant global health challenge, with thyroid cancer being one of the most prevalent endocrine malignancies. Despite advances in diagnosis and treatment, the prognosis for thyroid cancer patients, particularly those with advanced stages, remains poor, underscoring the urgent need for novel biomarkers and therapeutic targets. The pathogenesis of thyroid cancer involves intricate molecular mechanisms that are yet to be fully elucidated. Understanding these mechanisms is crucial for improving early diagnosis, assessing prognosis, and developing more effective treatment strategies [1,2,3,4,5].

Recent studies have highlighted the potential roles of various genes in the development and progression of thyroid cancer. Among these, *GZMK*, *TREM2*, and *OR4D10* have emerged as promising candidates due to their involvement in immune regulation and cellular processes that are often dysregulated in cancer. *GZMK*, a granzyme family member, has been implicated in immune surveillance and tumor cell killing [6,7]. *TREM2*, a triggering receptor expressed on myeloid cells, plays a role in immune cell activation and phagocytosis [8,9]. *OR4D10*, an olfactory receptor, although less studied in cancer, has been suggested to have functions beyond olfaction, including potential roles in cell proliferation and migration [10]. However, the specific contributions of these genes to thyroid cancer pathogenesis, as well as their potential as diagnostic and prognostic biomarkers, remain largely unknown.

In this study, we aimed to comprehensively investigate the expression patterns, prognostic significance, and functional roles of *GZMK*, *TREM2*, and *OR4D10* in thyroid cancer. By analyzing large-scale pan-cancer datasets, including The Cancer Genome Atlas (TCGA) and the Genotype-Tissue Expression (GTEx) project, we sought to determine the differential expression of these genes in thyroid cancer compared to normal tissues. Furthermore, we explored the association between their expression levels and patient prognosis, as well as their correlation with immune cell infiltration and oncogenic pathways. Our findings not only provide insights into the molecular mechanisms underlying thyroid cancer but also identify potential novel biomarkers and therapeutic targets for this disease. Through this research, we hope to contribute to the development of more effective strategies for the diagnosis, prognosis, and treatment of thyroid cancer.

## 2. Results

### 2.1. Pan-Cancer GZMK, TREM2, and OR4D10 Expression Analysis

**Figure 1:**
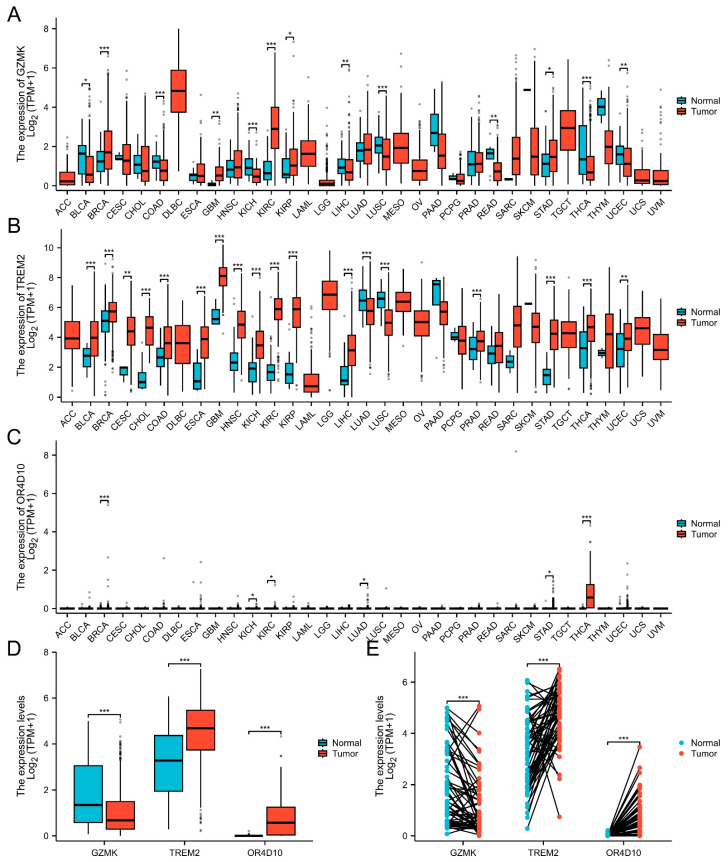
Pan-cancer *GZMK*, *TREM2* and *OR4D10* expression analysis. (**A**–**C**) *GZMK*, *TREM2*, and *OR4D10* expression in tumor and normal tissues in pan-cancer data of The Cancer Genome Atlas (TCGA) and GTEx. (**D**) *GZMK*, *TREM2*, and *OR4D10* expression in tumor and normal tissues in THCA from TCGA. (**E**) *GZMK*, *TREM2*, and *OR4D10* expression in paired tumor and normal tissues in THCA from TCGA. Data were shown as mean ± SD. * *p* < 0.05, ** *p* < 0.01, *** *p* < 0.001.

First, we looked at the expression of *GZMK*, *TREM2*, and *OR4D10* in the pan-cancer datasets GTEx and TCGA. Eight cancers were found to express *GZMK* less than the comparable normal tissues, such as BLCA, COAD, KICH, LIHC, LUSC, READ, THCA, and UCEC; sixteen tumors had higher *TREM2* expression than the corresponding normal tissues, such as BLCA, BRCA, CESC, CHOL, COAD, ESCA, GBM, HNSC, KICH, KIRC, KIRP, LIHC, PRAD, STAD, THCA, and UCEC; and six cancers expressed more *OR4D10* than the matching normal tissues, including BRCA, KICH, KIRC, LUAD, STAD, and THCA (Figure 1A–C).

In particular, THCA thyroid cancer in the TCGA cohort had lower *GZMK*, higher *TREM2*, and higher *OR4D10* expression (Figure 1D) compared to the adjacent tissues (Figure 1E). This suggests that *GZMK*, *TREM2*, and *OR4D10* may be involved in the pathogenesis of thyroid cancer.

### 2.2. Association Between GZMK, TREM2, and OR4D10 Expression and Cancer Patient Prognosis

**Figure 2:**
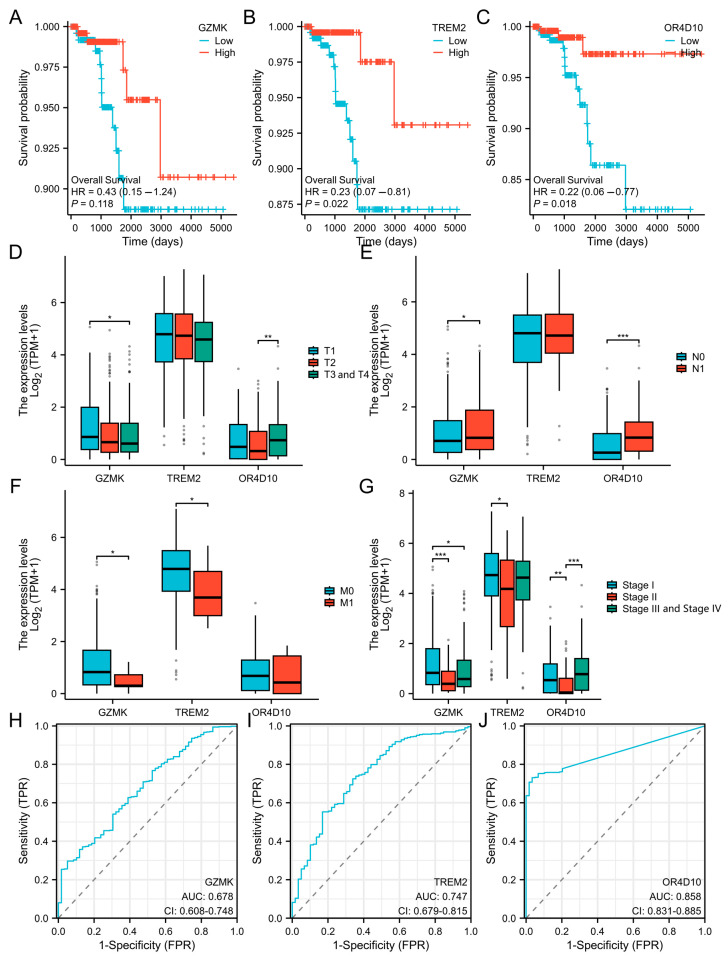
The association between *GZMK*, *TREM2*, *OR4D10* expression and cancer patient prognosis. (**A**–**C**) The correlation between *GZMK*, *TREM2*, and *OR4D10* expression and the prognosis of thyroid cancer was analyzed using The Cancer Genome Atlas (TCGA) database. (**D**–**G**) The clinical significance of different clinical variables in TCGA database. (**H**–**J**) The diagnostic efficacy of *GZMK*, *TREM2*, and *OR4D10*. * *p* < 0.05, ** *p* < 0.01, *** *p* < 0.001.

To assess the usefulness of *GZMK*, *TREM2*, and *OR4D10* expression in predicting the prognosis of cancer patients, we examined the relationship between *GZMK*, *TREM2*, and *OR4D10* expression and overall survival in the TCGA cohort. For the survival analyses, patients were stratified based on the median expression levels of *GZMK*, *TREM2*, and *OR4D10*, respectively. This stratification allowed us to compare the survival outcomes between patients with high and low expression levels of these genes. The results demonstrated that a poor prognosis in THCA was substantially correlated with reduced expression of *GZMK*, *TREM2*, and *OR4D10* (*p* = 0.041, 0.024, and 0.017) (Figure 2A–C), suggesting that *GZMK*, *TREM2*, and *OR4D10* are the potential oncogenes in this cancer.

In addition, to rule out the interference of confounding variables, we conducted a detailed comparison of the survival status among various subgroups. After meticulous analysis, it was found that across different groups, individuals with high expression of *GZMK*, *TREM2*, and *OR4D10* tended to have a higher survival rate, with *p*-values of 0.049, 0.026, 0.044, 0.006, and 0.046 (Supplementary Figure S1A–E).

*TREM2* was linked to M staging and pathological staging, *OR4D10* was linked to T, N, and pathological staging, and *GZMK* was linked to T, N, M, and pathological staging, according to our analysis of the expression of these three proteins in the development of thyroid cancer (Figure 2D–G). This suggests that the development of thyroid cancer is linked to *GZMK*, *TREM2*, and *OR4D10*.

Receiver Operating Characteristic (ROC) analysis was conducted to evaluate the diagnostic effectiveness of *GZMK*, *TREM2*, and *OR4D10* in distinguishing between normal and tumor groups. The AUC values for *GZMK*, *TREM2*, and *OR4D10* were 0.678 (*p* < 0.05), 0.747 (*p* < 0.05), and 0.858 (*p* < 0.05), respectively (Figure 2H–J). Based on the ROC analysis, the optimal cutoff values for *GZMK*, *TREM2*, and *OR4D10* were determined to be 0.3492, 3.8821, and 0.0548, respectively. At these cutoff values, the sensitivity, specificity, and diagnostic accuracy for *GZMK* were 30%, 95%, and 36%, respectively; for *TREM2*, they were 72%, 66%, and 72%, respectively; and for *OR4D10*, they were 73%, 97%, and 75%, respectively. These results demonstrate the diagnostic accuracy of *GZMK*, *TREM2*, and *OR4D10* in differentiating between normal and tumor groups.

### 2.3. Correlation and Enrichment Analyses

**Figure 3:**
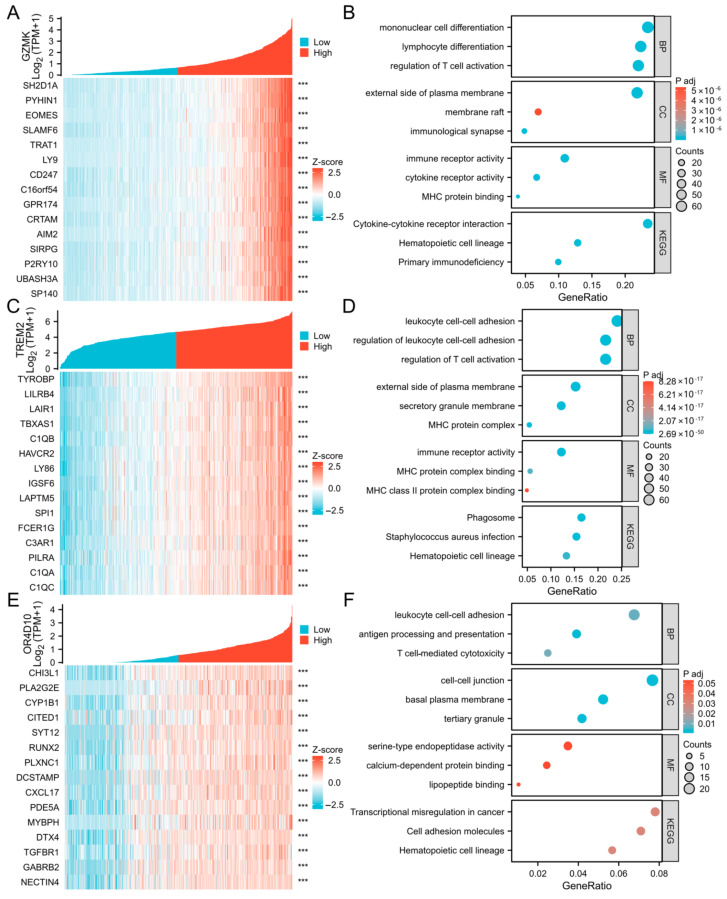
The correlation analysis of *GZMK*, *TREM2*, and *OR4D10*. (**A**,**C**,**E**) The top 15 genes most positively associated with *GZMK*, *TREM2*, and *OR4D10* were shown in a heatmap. Data were normalized by the Z-score standardization method. (**B**,**D**,**F**) Significant Gene Ontology terms of the top 300 genes most positively associated with *GZMK*, *TREM2*, and *OR4D10*, including biological processes, cell component, and molecular function. *** *p* < 0.001.

**Figure 4:**
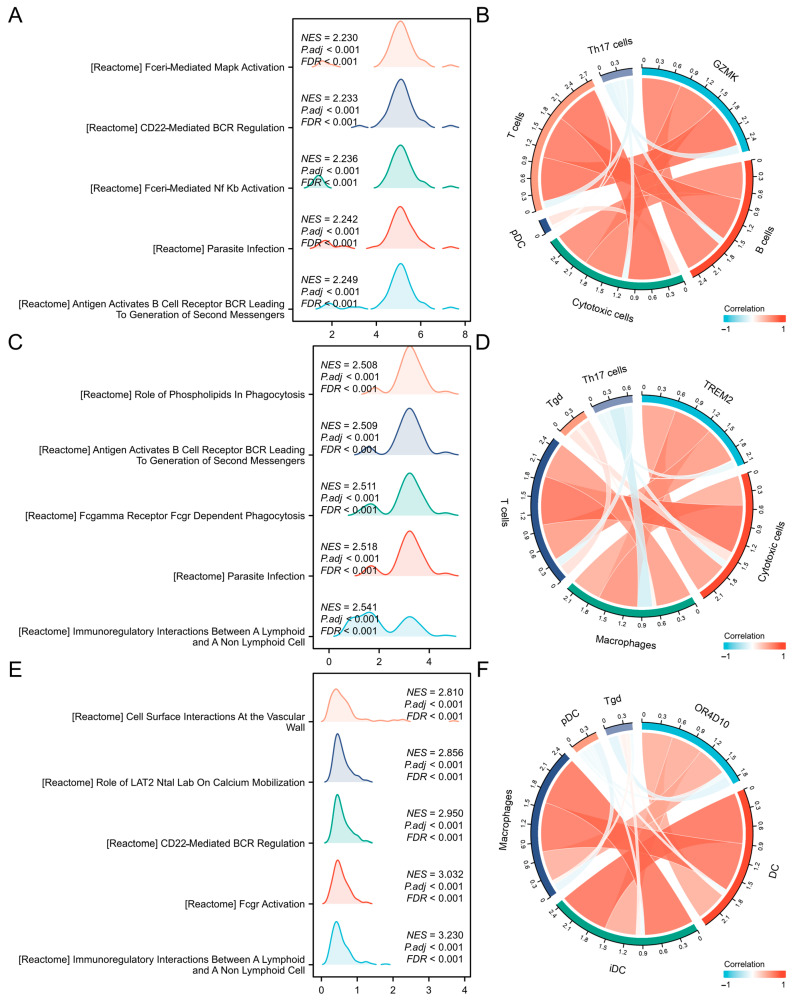
Enrichment analysis of *GZMK*, *TREM2* and *OR4D10* in thyroid cancer. (**A**,**C**,**E**) Significant gene set enrichment analysis (GSEA) results of *GZMK*, *TREM2*, and *OR4D10*, including KEGG pathways and Reactome pathways. (**B**,**D**,**F**) The correlation between *GZMK*, *TREM2*, *OR4D10*, and the immune cell infiltration levels; red represents a positive correlation, green represents a negative correlation, and the deeper the color, the stronger the correlation. Data were shown as mean ± SD.

To further understand the functions and pathways influenced by *GZMK*, *TREM2*, *OR4D10*, and all other mRNAs in thyroid cancer, we performed correlation studies using TCGA data. The top 15 genes were shown in a heatmap (Figure 3A,C,E), and the 300 genes most strongly linked to *GZMK*, *TREM2*, and *OR4D10* were chosen for enrichment analysis. Using the clusterProfiler program in R software(4.2.1), we also looked into potential functional pathways using the top 300 genes. *GZMK*, *TREM2*, and *OR4D10* were mostly linked to leukocyte cell–cell adhesion and mononuclear cell differentiation, according to GO functional enrichment analysis (Figure 3B,D,F). Gene set enrichment analysis was used to search the Reactome pathway and Kyoto Encyclopedia of Genes and Genomes (KEGG) databases. The vertical axis represents the name of the gene set, and the horizontal axis represents the distribution of values corresponding to genes in the core enrichment of the corresponding gene set. Phospholipids’ function in phagocytosis, Fceri-mediated mapk activation, and cell surface contacts at the vascular wall were all significantly enriched, according to the data (Figure 4A,C,E). The findings imply that elevated expression of *GZMK*, *TREM2*, and *OR4D10* is linked to the hyperactivation of several oncogenic pathways in thyroid cancer, including those that regulate cell division.

### 2.4. Correlation Between Immune Cell Infiltration and GZMK, TREM2, OR4D10 Expression

**Figure 5:**
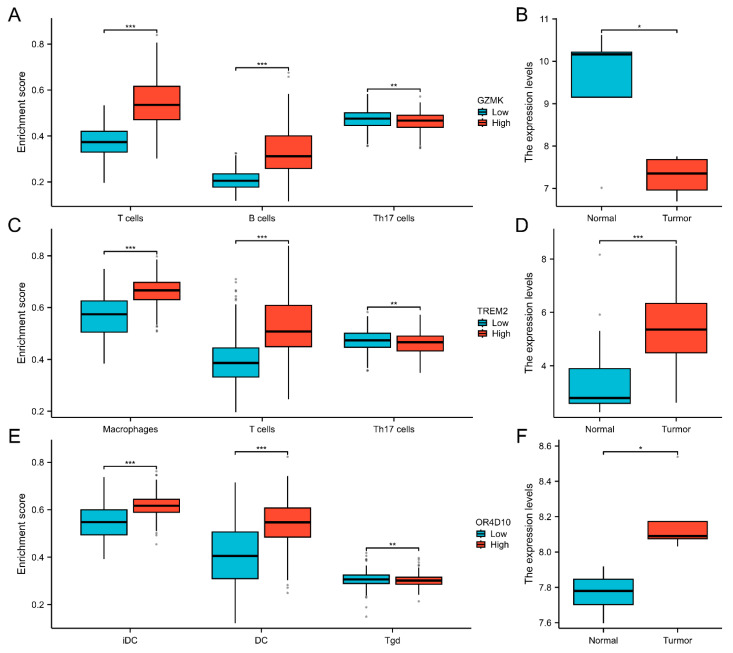
The correlation analysis between immune cell infiltration and *GZMK*, *TREM2*, *OR4D10* in thyroid cancer. (**A**,**C**,**E**) The immune cell infiltration level in groups of high and low *GZMK*, *TREM2*, and *OR4D10* expression in thyroid cancer of The Cancer Genome Atlas (TCGA) cohort. (**B**,**D**,**F**) The differential expression of *GZMK*, *TREM2*, and *OR4D10* in thyroid cancer in the GEO database. * *p* < 0.05, ** *p* < 0.01, *** *p* < 0.001.

We next examined the TCGA database’s immune cell infiltration ratings for thyroid cancer patients. T cell and DC infiltration scores were greater compared to the low-expression group in the high-expression group (Figure 5A,C,E).

T cells and DC infiltration were substantially positively connected with *GZMK*, *TREM2*, and *OR4D10* expression (Figure 4B,D,F), suggesting that elevated *GZMK*, *TREM2*, and *OR4D10* expression encourages intratumoral accumulation of T cells and DC. These findings imply a strong correlation between the immune-activated state of thyroid cancer and elevated expression of *GZMK*, *TREM2*, and *OR4D10*.

### 2.5. Validation of Gene Expression in GEO Database

This image displays a box and dot plot of the *GZMK*, *TREM2*, and *OR4D10* gene expression distributions in tumor and normal tissues. The y-axis shows the gene’s expression distribution, while the x-axis shows the various sample groups. Different groupings are represented by different colors. * denotes *p* < 0.05, ** denotes *p* < 0.01, and *** denotes *p* < 0.001, while the asterisks in the top left corner signify the significant *p*-value. The degree of relevance is indicated by the number of asterisks. The t-test or Wilcoxon test was used to assess whether the difference between two sample groups was statistically significant (Figure 5B,D,F).

We conducted a comprehensive validation of the enrichment analysis by utilizing the GEO database. Specifically, in the dataset GSE50901, our findings revealed a significant correlation: high expression levels of *GZMK* were linked to mononuclear cell differentiation, while elevated expression of *TREM2* was associated with leukocyte cell–cell adhesion processes (Supplementary Figure S2A,B).

## 3. Discussion

The current study presents a comprehensive analysis of the expression patterns and potential roles of *GZMK*, *TREM2*, and *OR4D10* in pan-cancer, with a particular focus on thyroid cancer (THCA). Our findings reveal significant differences in the expression of these genes between tumor and normal tissues across multiple cancer types. Specifically, in THCA, we observed lower *GZMK* and higher *TREM2* and *OR4D10* expression in tumor tissues compared to adjacent normal tissues, suggesting their involvement in thyroid cancer pathogenesis. These results are consistent with previous studies that have implicated these genes in immune regulation and cancer progression, although their specific roles in thyroid cancer were previously unclear [11,12].

Furthermore, our analysis of the association between *GZMK*, *TREM2*, and *OR4D10* expression and cancer patient prognosis in the TCGA cohort demonstrated that reduced expression of *GZMK* and increased expression of *TREM2* and *OR4D10* were significantly correlated with poor prognosis in THCA. This suggests that these genes may serve as potential prognostic markers or therapeutic targets in thyroid cancer. Additionally, our correlation and enrichment analyses revealed that *GZMK*, *TREM2*, and *OR4D10* are involved in leukocyte cell–cell adhesion, mononuclear cell differentiation, and the regulation of oncogenic pathways, such as phospholipids’ function in phagocytosis and cell surface contacts at the vascular wall. These findings provide new insights into the molecular mechanisms underlying thyroid cancer progression and suggest that these genes may play important roles in the tumor microenvironment.

In our study, *GZMK*, *TREM2*, and *OR4D10* showed varying diagnostic effectiveness. *GZMK* had low sensitivity (30%) and diagnostic accuracy (36%) despite high specificity (95%). *TREM2* and *OR4D10* had better overall performance, with *OR4D10* showing the highest specificity (97%) and good accuracy (75%). The low sensitivity and accuracy of *GZMK* as a diagnostic marker warrant further investigation to understand its limitations.

Interestingly, our analysis of immune cell infiltration in thyroid cancer patients showed that high expression of *GZMK*, *TREM2*, and *OR4D10* was associated with increased T cell and DC infiltration, indicating a strong correlation between the immune-activated state of thyroid cancer and elevated expression of these genes. This is consistent with previous studies that have shown that immune cell infiltration is a critical factor in cancer progression and response to therapy [13,14,15,16,17]. Our findings suggest that *GZMK*, *TREM2*, and *OR4D10* may play important roles in modulating the immune response in thyroid cancer, potentially through their effects on leukocyte cell–cell adhesion and mononuclear cell differentiation.

In conclusion, our study provides new insights into the expression patterns and potential roles of *GZMK*, *TREM2*, and *OR4D10* in pan-cancer, with a particular focus on thyroid cancer. Our findings suggest that these genes may serve as potential prognostic markers or therapeutic targets in thyroid cancer and may play important roles in modulating the immune response in the tumor microenvironment. Future studies are needed to further elucidate the molecular mechanisms underlying the effects of these genes in thyroid cancer and to explore their potential as therapeutic targets. Additionally, it would be interesting to investigate the potential interactions between *GZMK*, *TREM2*, and *OR4D10* and other immune-related genes in the tumor microenvironment, which could provide new insights into the complex interplay between cancer cells and the immune system.

## 4. Materials and Methods

### 4.1. Data Collection and Analysis

The University of California, Santa Cruz (UCSC) Xena database (https://xenabrowser.net/datapages/, accessed on 1 February 2025) was used to download *GZMK*, *TREM2*, and *OR4D10* expression profiles as well as clinical pan-cancer data using TCGA and Genotype-Tissue Expression (GTEx). These included information on adrenal cortical carcinoma, bladder urothelial carcinoma, breast cancer invasive carcinoma, cervical squamous cell carcinoma and endometrial adenocarcinoma, cholangiocarcinoma, colon adenocarcinoma, esophageal carcinoma, polymorphic glioblastoma, head and neck squamous cell carcinoma, renal chromophobe cell carcinoma, renal clear cell carcinoma, renal cell carcinoma, acute myeloid leukemia, brain low-grade glioma, hepatocellular carcinoma, lung adenocarcinoma, lung squamous cell carcinoma, ovarian serous cystadenocarcinoma, pancreatic cancer, prostate adenocarcinoma, rectal adenocarcinoma, skin melanoma, gastric adenocarcinoma, testicular germ cell tumor, thyroid cancer, uterine endometrial carcinoma, and uterine carcinosarcoma. *GZMK*, *TREM2*, and *OR4D10* expression were measured in tumor and normal tissues obtained from the TCGA and GTEx databases, respectively. (ACC for adrenocortical carcinoma, BLCA for bladder urothelial carcinoma, BRCA for breast invasive carcinoma, CESC for cervical squamous cell carcinoma and endocervical adenocarcinoma, CHOL for cholangiocarcinoma, COAD for colon adenocarcinoma, COADREAD for colon and rectum adenocarcinoma, DLBC for diffuse large B cell lymphoma, ESCA for esophageal carcinoma, GBM for glioblastoma multiforme, GBMLGG for glioma, HNSC for head and neck squamous cell carcinoma, KICH for kidney chromophobe, KIRC for kidney renal clear cell carcinoma, KIRP for kidney renal papillary cell carcinoma, LAML for acute myeloid leukemia, LGG for brain lower-grade glioma, LIHC for liver hepatocellular carcinoma, LUAD for lung adenocarcinoma, LUSC for lung squamous cell carcinoma, MESO for mesothelioma, OV for ovarian serous cystadenocarcinoma, PAAD for pancreatic adenocarcinoma, PCPG for pheochromocytoma and paraganglioma, PRAD for prostate adenocarcinoma, READ for rectum adenocarcinoma, SARC for sarcoma, SKCM for skin cutaneous melanoma, STAD for stomach adenocarcinoma, STES for stomach and esophageal carcinoma, TGCT for testicular germ cell tumors, THCA for thyroid carcinoma, THYM for thymoma, UCEC for uterine corpus endometrial carcinoma, UCS for uterine carcinosarcoma, and UVM for uveal melanoma.)

### 4.2. Correlation and Enrichment Analyses

*GZMK*, *TREM2*, *OR4D10*, and other mRNAs were subjected to Pearson correlation analysis in thyroid cancer using TCGA THCA data. The function of *GZMK*, *TREM2*, and *OR4D10* was ascertained by conducting an enrichment analysis on the 300 genes that were most positively correlated with these three proteins. The clusterProfiler R software[4.4.4] package’s EnrichGO function was used to conduct gene ontology (GO) analysis with the subsequent configurations: *p*-value-cutoff = 0.05, q-value-cutoff = 0.05, and ont = all. Using clusterProfiler’s gseKEGG and gsePathway functions, gene set enrichment analysis was carried out using the following settings: Perm = 1000, *p*-value-cutoff = 0.05, minGSSize = 10, and maxGSSize = 1000.

### 4.3. Immune Cell Infiltration

From a previously published study, we extracted pan-cancer immune cell infiltration ratings from TCGA using the CIBERSORT analytic tool. The median expression levels of *GZMK*, *TREM2*, and *OR4D10* (high versus low) were used to compare the immune cell infiltration levels of TCGA thyroid cancer samples across two groups.

### 4.4. Statistical Analyses

The means ± standard deviation are used to express the data. SPSS software, version 21.0 (SPSS, Inc., Chicago, IL, USA) was used to analyze all of the data. The Kolmogorov–Smirnov test was used to determine if the data were normal. Student’s *t*-test was used to examine group differences pairwise. To account for multiple comparisons, the false discovery rate (FDR) correction was applied to the *p*-values obtained from the statistical tests. A significant criterion of *p* < 0.05 was established.

## Data Availability

Online repositories include the datasets used in this investigation. The accession number or numbers and the names of the repository or repositories are included in the paper or Supplementary Materials.

## Supplementary Materials

The following supporting information can be downloaded at: https://www.mdpi.com/article/10.3390/ijms26083887/s1.
