# Supplementary material for: Upregulation of GZMK, TREM2, and OR4D10 as Prognostic Biomarkers in Thyroid Cancer: A Pan-Cancer and TCGA Data Analysis
Source: Int J Mol Sci. 2025 Apr 20;26(8):3887. doi: 10.3390/ijms26083887 (PMC12027860; doi:10.3390/ijms26083887)
Supplement: Supplementary file 1 [file ijms-26-03887-s001.zip › ijms-3556773-supplementary.pdf]

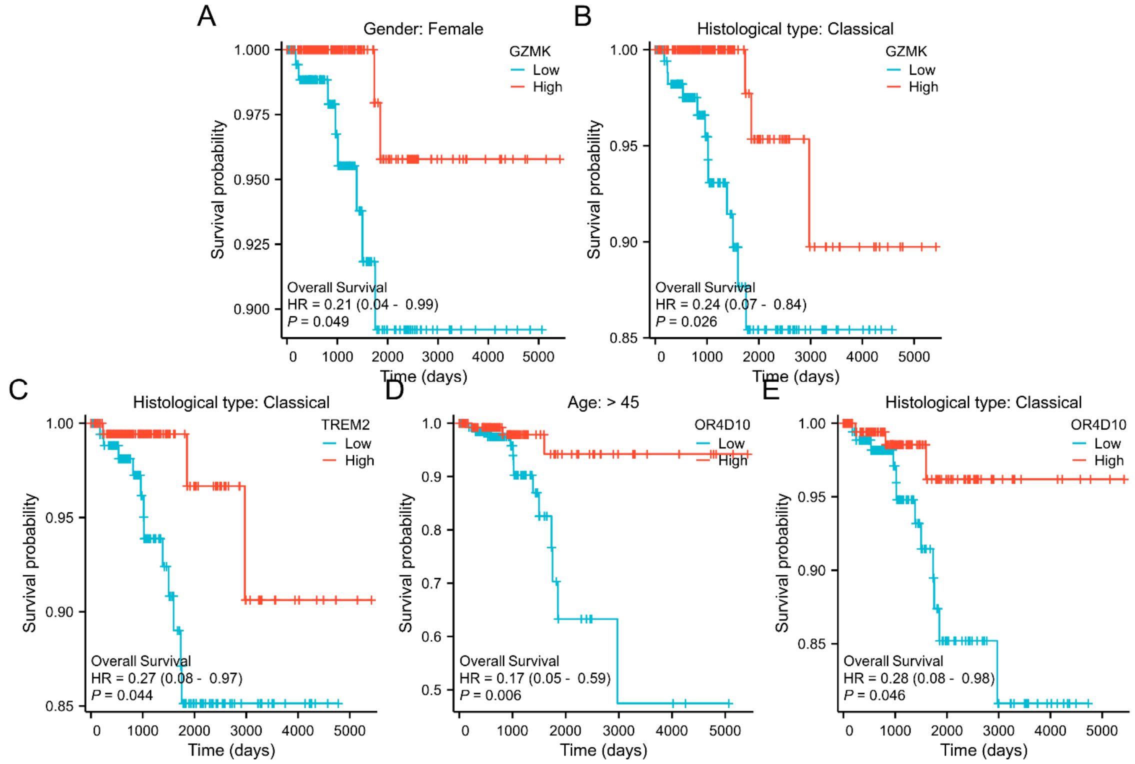

Figure S1. The association between GZMK, TREM2, OR4D10 expression in different subgroups and cancer patient prognosis. (A–E) The correlation between GZMK, TREM2 and OR4D10 expression and the prognosis of thyroid cancer was analyzed using The Cancer Genome Atlas (TCGA) database.

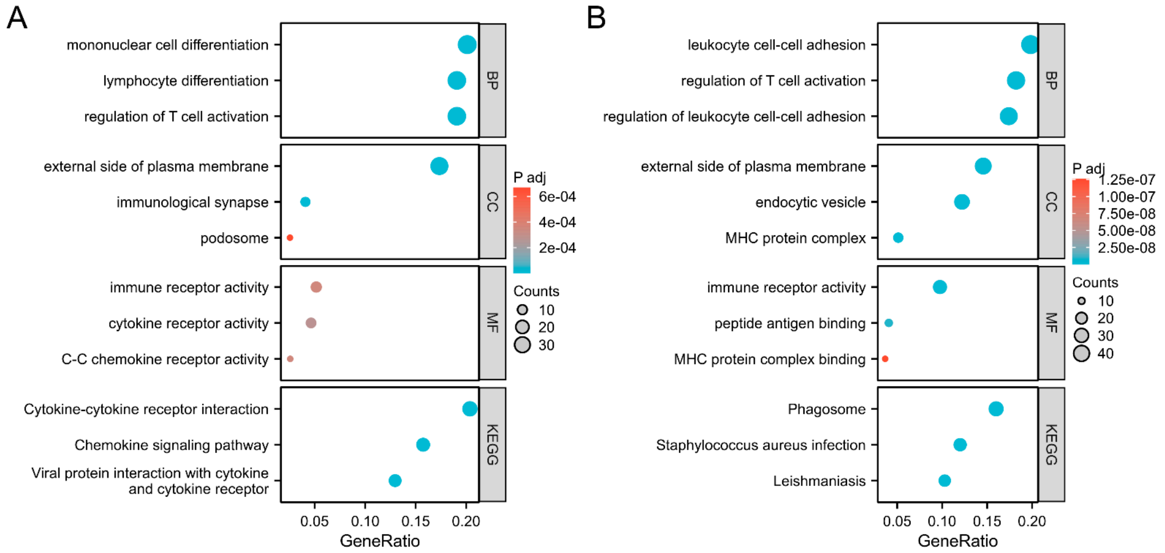

Figure S2. Enrichment analysis in GEO database. (A, B) Significant Gene Ontology terms of top 300 genes most positively associated with GZMK and TREM2, including biological processes, cell component, and molecular function.
